# Supplementary material for: The role of TORC1 in muscle development in Drosophila
Source: Sci Rep. 2015 Apr 13;5:9676. doi: 10.1038/srep09676 (PMC4394354; doi:10.1038/srep09676)
Supplement: Supplementary Information [file srep09676-s1.pdf]

The role of TORC1 in muscle development in Drosophila.

Isabelle Hatfield, Innocence Harvey, Erika R. Yates, JeAnna R. Redd, Lawrence T. Reiter and Dave Bridges

Supplementary Data

Supplementary Figure 1: Dose response of rapamycin on fly eclosure and larvae development. Flies were mated in the presence of varying doses of rapamycin. After 7 days, the mated adults were removed. After 21 days, the number of progeny and pupal cases were counted.

Supplementary Figure 2: Effects of lowered GAL4 expression on eclosure of *24B*-GAL4 driven *Raptor* knockdowns. The graph depicts the relative birth rates of the progeny produced when the *24B*-GAL4>UAS-*Raptor*-shRNA crosses were repeated at 18° C. The double balancer genotype is excluded from the graph. The dashed line represents the expected birth rates of the three genotypes. The colder environment was unable to rescue the lethality effect of *Raptor* knockdown in skeletal muscle.

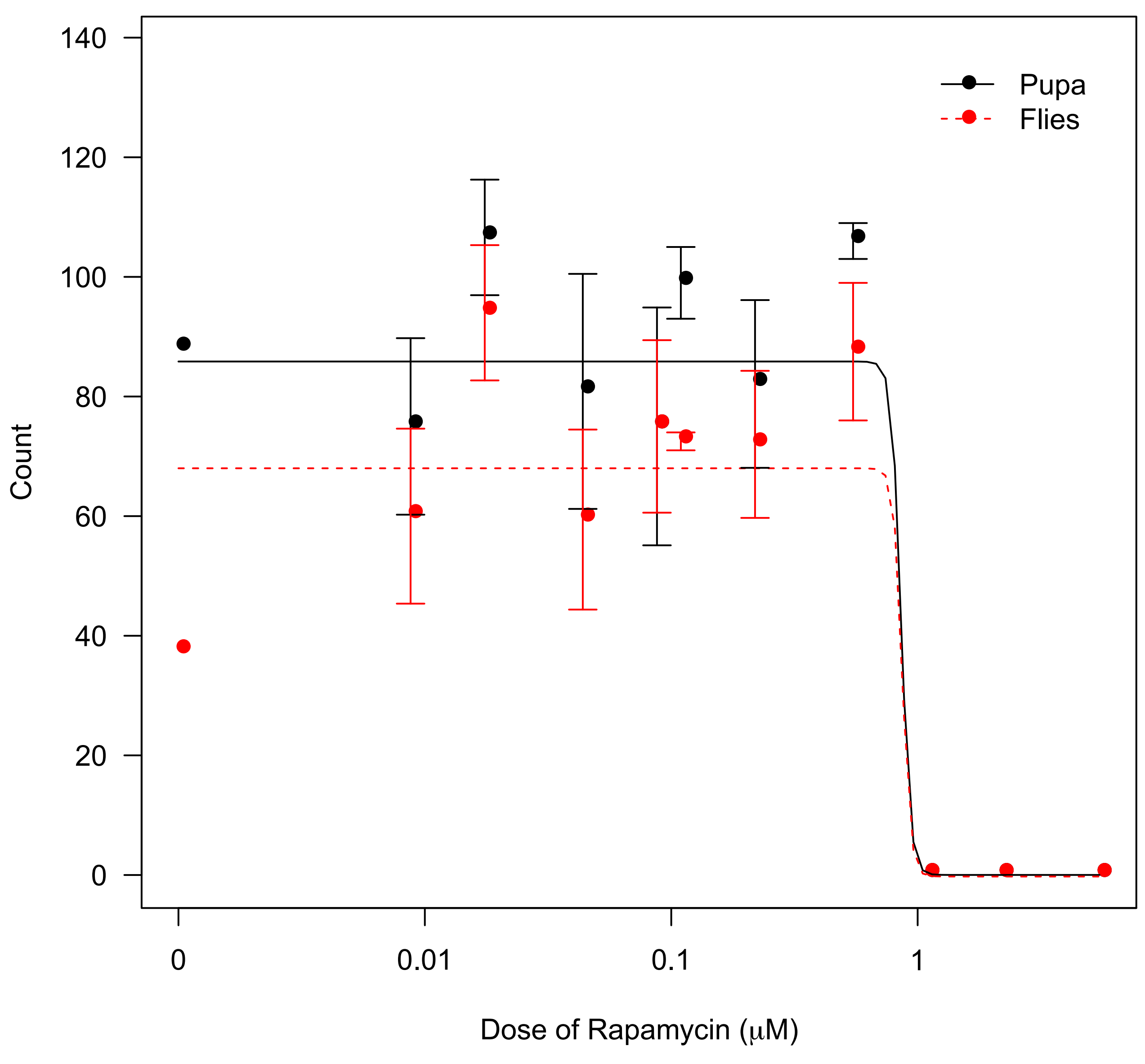

Pupa
Flies
Count
140
120
100
80
60
40
20
0
0
0.01
0.1
1
Dose of Rapamycin (μM)

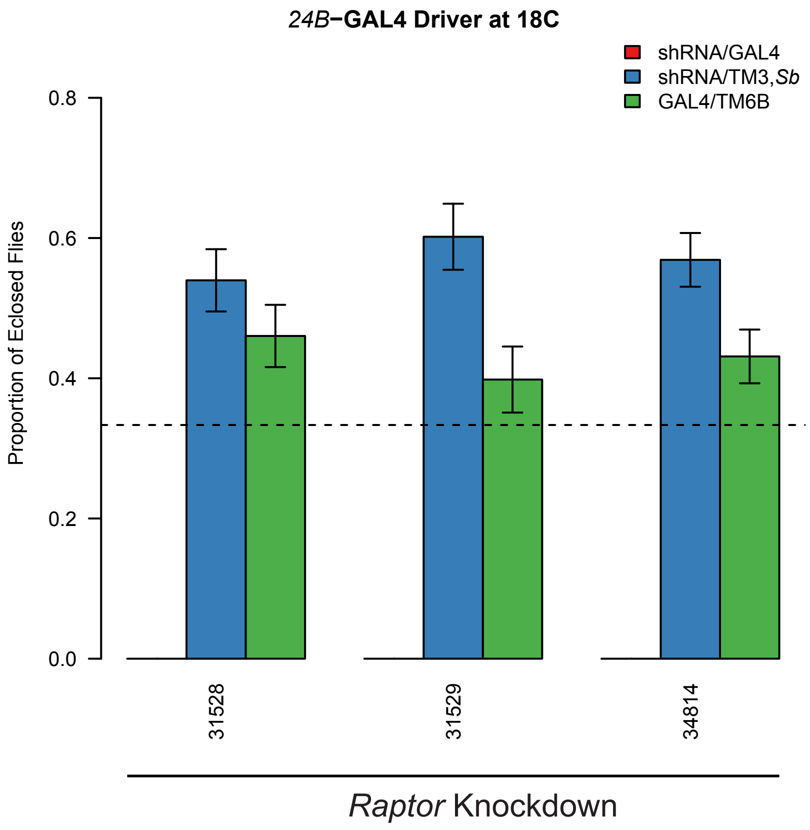

24B−GAL4 Driver at 18C
shRNA/GAL4
shRNA/TM3,Sb
GAL4/TM6B
Proportion of Eclosed Flies
0.0
0.2
0.4
0.6
0.8
31528
31529
34814
Raptor Knockdown
